# Red Cell Distribution Width-Coefficient of Variation and Its Correlation With Components of Metabolic Syndrome: A Case-Control Study

**DOI:** 10.7759/cureus.113779

**Published:** 2026-08-01

**Authors:** Akash Bharti, Praveen R Mishra, Shailly Singhania, Aditya Chakravorty, Manuj Shukla, VPS Punia

**Affiliations:** 1 Internal Medicine, North Delhi Municipal Corporation Medical College, Delhi, IND; 2 Internal Medicine, School of Medical Sciences and Research, Greater Noida, IND; 3 Nephrology, Fortis Hospital Vasant Kunj, New Delhi, IND

**Keywords:** cardiovascular risk, dyslipidemia, inflammation, mets, oxidative stress, rdw-cv

## Abstract

Background: Changes in erythrocyte shape caused by oxidative stress and persistent low-grade inflammation associated with metabolic syndrome (MetS) might cause variations in red cell distribution width-coefficient of variation (RDW-CV). This study assessed RDW-CV in MetS patients and looked at how it related to biochemical and clinical indicators. Changes in erythrocyte shape caused by oxidative stress and persistent low-grade inflammation associated with MetS might cause variations in RDW-CV.

Methods: This case-control study was conducted in the general medicine department of Sharda Hospital in Greater Noida between January 2020 and June 2021. Fifty patients between the ages of 18 and 60 who satisfied the diagnostic criteria for MetS were included, along with fifty age- and sex-matched healthy controls. The clinical evaluation included blood pressure checks and anthropometric measurements. Laboratory examinations included complete blood counts (including RDW-CV), fasting and postprandial blood glucose, liver function tests, renal function tests, lipid profiles, and thyroid function testing.

Results: Patients with metabolic syndrome exhibited a significantly higher mean RDW (15.06±1.64% vs. 13.22±1.29%) than healthy controls. RDW-CV showed strong associations with high-density lipoprotein (HDL) cholesterol, serum triglyceride levels, and abdominal circumference. RDW-CV quartiles, however, did not appear to be correlated with any other clinical variables. Greater RDW-CV quartiles were associated with a reduced hazard of metabolic syndrome in male individuals.

Conclusion: The mean RDW-CV was significantly greater in patients with MetS, and there were strong correlations with HDL cholesterol, triglycerides, and abdominal circumference. These findings suggest that RDW-CV may be a conveniently available and reasonably priced hematological marker that reflects the metabolic and inflammatory changes linked to metabolic syndrome. To confirm its clinical usefulness, larger prospective investigations are necessary.

## Introduction

Insulin resistance, obesity, hypertension, increased triglycerides (TG), and low levels of high-density lipoprotein (HDL) cholesterol are all part of metabolic syndrome (MetS). According to recent research, issues associated with obesity may be significantly influenced by free fatty acids produced from adipose tissue. Systemic oxidative stress is thought to be a major factor, even though the precise processes by which adipose tissue growth contributes to metabolic dysregulation are yet unclear [1,2].

According to a number of studies, obesity causes oxidative stress by increasing the formation of reactive oxygen species (ROS) during fatty acid oxidation in mitochondria and peroxisomes. Additionally, this process produces malondialdehyde (MDA), a byproduct of lipid peroxidation that promotes the production of pro-inflammatory cytokines and adds to systemic oxidative stress. Red blood cell survival and homeostasis are negatively impacted by oxidative stress, and higher red cell distribution width (RDW) has been linked to lower serum antioxidant concentrations. RDW is a measure of variability in circulating erythrocyte (anisocytosis) size and is routinely reported as part of the complete blood count. RDW is expressed as two different indices: RDW-coefficient of variation (CV) and RDW-standard deviation (SD). RDW-CV is expressed as a percentage and is calculated as the standard deviation of red blood cell volume divided by the mean corpuscular volume (MCV); thus, it reflects erythrocyte size variability with respect to MCV. Red blood cell turnover may be accelerated by increased oxidative stress, which could lead to more erythrocyte size fluctuation (anisocytosis) and an increased RDW-CV [3-5].

Erythropoiesis is hampered by interleukin-1, pro-inflammatory cytokines, and interleukin-6, which suppress erythropoietin synthesis and activity. Therefore, elevated RDW may be a result of chronic inflammation, which is frequently seen in patients with metabolic syndrome and cardiovascular disease [4,5].

Atherosclerosis is caused by a number of metabolic problems, the most significant of which are dyslipidemia, chronic inflammation, and impaired coagulation. By hindering iron metabolism, reducing erythropoietin activity, and interfering with erythrocyte maturation, the low-grade inflammatory state linked to metabolic syndrome may encourage anisocytosis. Furthermore, inflammation reduces erythrocyte survival, which makes the population of red blood cells in circulation more diverse [4,5].

The goal of the current study was to assess RDW-CV in individuals with metabolic syndrome and ascertain its relationship to blood pressure, blood glucose levels, body mass index (BMI), waist circumference, and lipid profile.

## Materials and methods

Study design and setting

From January 2020 to June 2021, the Department of General Medicine at Sharda Hospital, School of Medical Sciences and Research, Greater Noida, was the site of this hospital-based case-control study.

Study population

The study included 50 patients diagnosed with metabolic syndrome and 50 apparently healthy age- and sex-matched controls. Participants were between 18 and 60 years of age. Patients were recruited from the Department of General Medicine after applying the predefined inclusion and exclusion criteria.

Sample size calculation

The sample size was calculated based on the study by Sánchez-Chaparro et al, which demonstrated a significant association between high red cell distribution width (RDW) and metabolic syndrome [6]. The minimum sample size was calculated to be 45 participants per group, assuming a 95% confidence level, 80% study power, and an expected moderate effect size based on the reported difference in RDW between the subjects with and without metabolic syndrome. The total sample size of 100 participants was enrolled in the study: 50 cases and 50 age- and sex-matched controls to compensate for possible exclusions and incomplete data.

Inclusion criteria

The study included patients between the ages of 18 and 60 who met the International Diabetes Federation's (IDF) diagnostic criteria for metabolic syndrome.

Exclusion criteria

Patients with any of the following conditions were excluded, i.e., autoimmune disorders, chronic kidney disease, malignancy, pregnancy, current corticosteroid therapy, hemoglobinopathies, anemia, bone marrow disorder, acute hemorrhage or recent major surgery (within three months), medication (iron, vitamin B12, and folic acid supplementation), erythropoietin-stimulating factors, high-dose corticosteroids. 

Diagnostic criteria for metabolic syndrome (MetS)

MetS was diagnosed according to the IDF criteria, which require central obesity together with any two of the following four components (Table 1).

**Table 1:** Diagnostic criteria for MetS. MetS: metabolic syndrome; HDL: high-density lipoprotein.

Component	Diagnostic criteria
Elevated triglycerides	Triglyceride level ≥150 mg/dL (1.7 mmol/L) or specific treatment for this lipid abnormality.
Reduced HDL cholesterol	Males: <40 mg/dL (1.03 mmol/L); Females: <50 mg/dL (1.29 mmol/L), or specific treatment for this lipid abnormality.
Elevated blood pressure	Systolic blood pressure (SBP) ≥130 mmHg and/or diastolic blood pressure (DBP) ≥85 mmHg, or treatment for previously diagnosed hypertension.
Elevated fasting plasma glucose	Fasting plasma glucose ≥100 mg/dL (5.6 mmol/L) or previously diagnosed type 2 diabetes mellitus.

According to the IDF recommendations, central obesity may be assumed in individuals with a BMI ≥30 kg/m²; therefore, measurement of waist circumference is not mandatory in such cases.

Clinical and laboratory evaluation

After obtaining each participant's complete medical history, a thorough physical examination was conducted. Blood pressure was measured using standard methods, and anthropometric data, such as waist circumference and BMI, were recorded. Following an overnight fast, venous blood samples were taken. Among the laboratory data recorded were total blood count (RDW-CV, erythrocyte sedimentation rate (ESR)), fasting blood glucose, blood glucose two hours after a meal, lipid profile, liver function tests, kidney function tests, and thyroid function tests.

Laboratory procedures

After an overnight fast of 8-12 hours, 5 mL of venous blood was collected under aseptic precautions from each individual. The complete blood count parameters, including RDW-CV, were measured by an automated hematology analyzer (Sysmex XN-550, Sysmex Corporation, headquartered in Kobe, Japan). Fasting and postprandial blood glucose levels were estimated by the glucose oxidase-peroxidase method using an automated biochemistry analyzer (Mindray BS-200, Shenzhen Mindray Bio-Medical Electronics Co., Ltd., China). Serum triglycerides, total cholesterol, HDL cholesterol, and other biochemical parameters were analyzed enzymatically using commercially available reagent kits on the same analyzer, according to the manufacturer's instructions. Standard laboratory procedures were used to carry out liver function tests, kidney function tests, and thyroid function tests in the central clinical laboratory of Sharda Hospital.

Statistical analysis

Data were analyzed using SPSS Version 22 (IBM Corp., Armonk, New York, USA). Categorical data were presented as frequency and percentage, whereas continuous variables were presented as mean±SD. To compare study groups, chi-square and t-tests were used. Pearson correlation was used to examine RDW-CV and clinical and biochemical variables. A p-value <0.05 indicated statistical significance.

## Results

The mean age among metabolic syndrome patients was 47.66±12.05 years, and among age-matched controls, 42.58±15.62 years. Patients with metabolic syndrome had significantly higher abdominal circumference, BMI, DBP, SBP, TG, serum glutamic oxaloacetic transaminase (SGOT), fasting blood sugar (FBS), serum glutamic pyruvic transaminase (SGPT), and serum uric acid levels compared with controls. Hemoglobin and thyroid-stimulating hormone (TSH) levels were similar between the two groups (Table 2).

**Table 2:** Baseline characteristics. *Statistically significant.

Variable	Reference range (adults)	Metabolic syndrome (n = 50) Mean±SD	Age matched controls (n = 50) Mean±SD	p-value
Age		47.66±12.05	42.58±15.62	0.24
Waist circumference	Men: <90 cm; Women: <80 cm (South Asians)	100.69±13.24	72.82±8.77	<0.01*
Body mass index	18.5-24.9 kg/m²	30.08±4.31	22.09±1.92	<0.01*
Systolic blood pressure	<120 mmHg	137.28±16.45	117.20±11.24	<0.01*
Diastolic blood pressure	<80 mmHg	87.40±8.59	72.44±8.83	<0.01*
Fasting blood sugar	70-99 mg/dL	148.18±67.07	96.22±18.05	<0.01*
Triglycerides	<150 mg/dL	214.18±103.17	135.70±22.54	<0.01*
High-density lipoprotein cholesterol	Men: ≥40 mg/dL; Women: ≥50 mg/dL	37.66±4.26	49.34±12.17	<0.01*
Hemoglobin	Men: 13.5-17.5 g/dL; Women: 12.0-15.5 g/dL	13.23±1.19	13.26±0.97	0.87
Serum glutamic oxaloacetic transaminase (aspartate aminotransferase; AST)	10-40 U/L	55.68±35.27	38.17±15.64	0.002*
Serum glutamic pyruvic transaminase (alanine aminotransferase; ALT)	7-56 U/L	59.54±41.09	41.78±22.58	0.009*
Serum uric acid	Men: 3.4-7.0 mg/dL; Women: 2.4-6.0 mg/dL	5.63±3.85	4.31±1.85	0.031*
Thyroid-stimulating hormone	0.4-4.0 mIU/L	3.52±2.34	3.36±1.92	0.73
Gender, n (%) Male: Female		24 (48): 26 (52)	23 (46): 27 (54)	0.84

**Figure 1:**
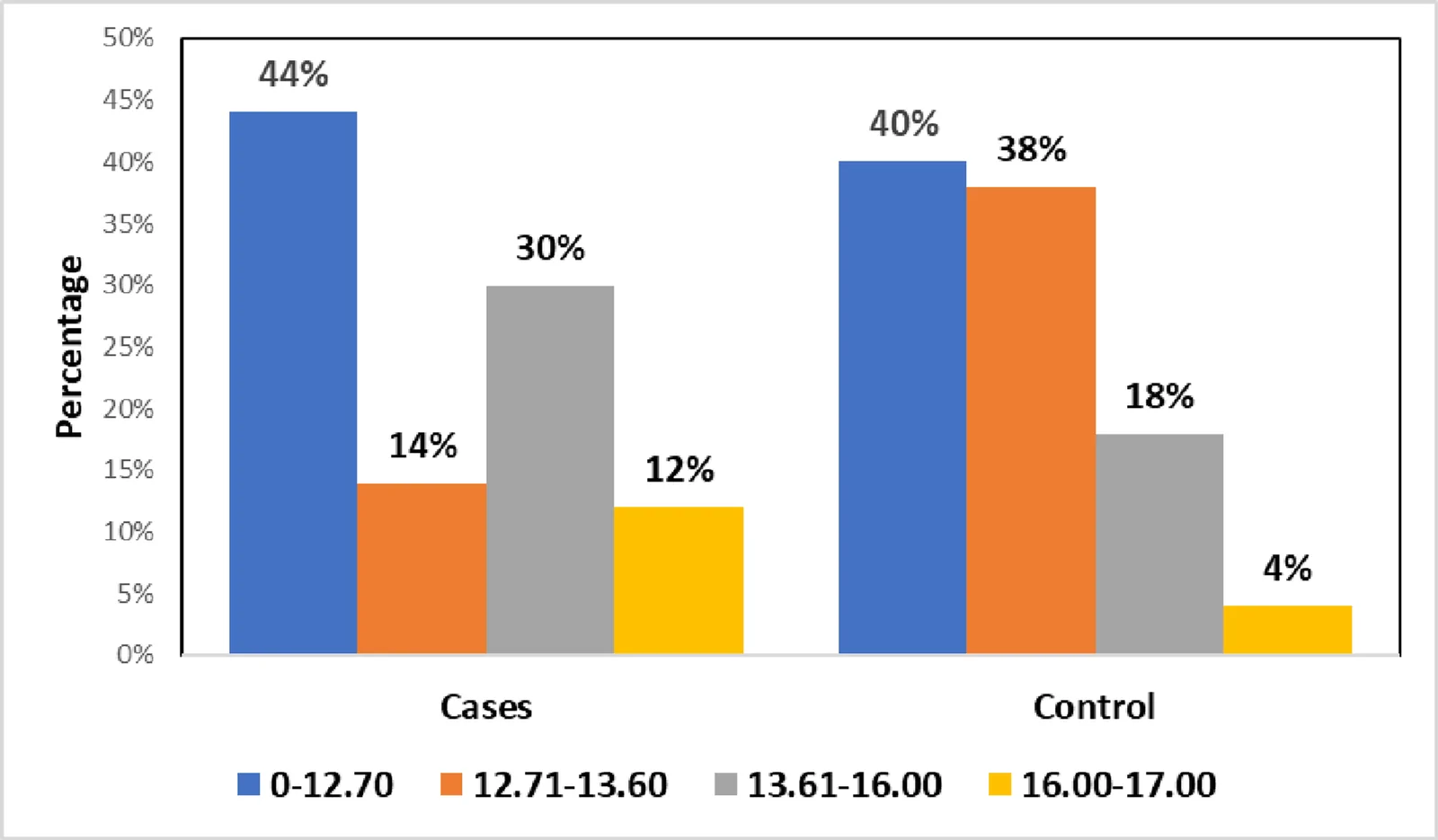
Distribution of RDW-CV quartiles. RDW-CV: red cell distribution width-coefficient of variation.

An RDW-CV value >14.5% was considered elevated, whereas values between 11.5% and 14.5% were considered within the normal range. The incidence of metabolic syndrome decreased significantly as the proportion of RDW-CV quartiles grew higher. The total mean RDW-CV among metabolic syndrome patients was 15.06±1.64, and among age-matched controls was 13.22±1.29 (Figure 1).

When patients were categorized into RDW-CV quartiles, none of the clinical or biochemical variables demonstrated statistically significant differences across quartiles (p > 0.05 for all comparisons), although a gradual increase in fasting blood glucose and triglyceride levels and a reduction in abdominal circumference were observed across higher quartiles (Table 3).

**Table 3:** Association between RDW-CV quartiles and clinical variables. RDW-CV: red cell distribution width-coefficient of variation.

Variable	RDW-CV quartiles				p-value
	Q1	Q2	Q3	Q4	
Age	38.33±11.53	54.43±8.58	51.07±13.46	45.73±10.65	0.051
Abdominal circumference	110.00±18.23	102.00±15.93	99.08±14.07	95.84±9.73	0.304
Body mass index	32.22±3.95	30.36±4.37	29.21±4.67	30.00±4.21	0.559
Systolic blood pressure	139.00±19.26	138.57±19.38	136.07±16.86	137.23±15.61	0.981
Diastolic blood pressure	87.67±7.09	82.57±5.85	87.60±9.51	88.73±8.97	0.443
Fasting blood sugar	129.00±55.45	149.14±69.73	144.93±71.58	155.32±69.23	0.863
Triglycerides	159.33±95.49	222.71±125.11	217.60±95.86	224.09±105.22	0.593
High-density lipoprotein cholesterol	54.18±13.59	45.29±9.23	44.04±14.55	52.95±9.49	0.083
Hemoglobin	12.80±0.61	13.10±1.38	13.45±1.17	13.24±1.31	0.73
Serum glutamic oxaloacetic transaminase (aspartate aminotransferase; AST)	30.00±10.23	30.51±7.33	40.48±21.77	41.27±12.74	0.214
Serum glutamic pyruvic transaminase (alanine aminotransferase; ALT)	28.83±13.41	31.11±10.56	47.18±28.26	45.04±21.58	0.184
Serum uric acid	4.93±1.72	5.09±1.93	4.90±1.51	6.48±5.48	0.598
Thyroid-stimulating hormone	2.98±1.55	5.19±2.63	3.19±2.79	3.35±1.97	0.232

RDW-CV and abdominal circumference and serum triglyceride levels showed a slight but statistically significant positive connection, according to bivariate correlation analysis. HDL cholesterol and RDW-CV had a slight negative connection (r = -0.30, p = 0.034). Age, BMI, blood pressure, fasting blood glucose, liver enzymes, serum uric acid, hemoglobin, and TSH did not significantly correlate with RDW (Table 4).

**Table 4:** Bivariate correlation analysis between clinical and biochemical variables and RDW-CV among patients with metabolic syndrome (N = 50). *Statistically significant. CC: correlation coefficient; RDW-CV: red cell distribution width-coefficient of variation.

Variable	CC	p-value
Age	0.23	0.11
Abdominal circumference	0.31	0.028*
Body mass index	-0.052	0.72
Systolic blood pressure	0.029	0.84
Diastolic blood pressure	0.225	0.17
Fasting blood sugar	0.167	0.25
Triglycerides	0.41	0.003*
High-density lipoprotein cholesterol	-0.30	0.034*
Hemoglobin	0.01	0.94
Serum glutamic oxaloacetic transaminase (aspartate aminotransferase; AST)	0.21	0.15
Serum glutamic pyruvic transaminase (alanine aminotransferase; ALT)	0.18	0.22
Serum uric acid	0.17	0.24
Thyroid-stimulating hormone	-0.116	0.42

Table 5 shows the distribution of RDW-CV quartiles according to gender among patients with metabolic syndrome. Male participants were more frequently represented in the lowest RDW-CV quartile (0-12.70%), whereas female participants predominated in the higher RDW-CV quartiles, particularly in the highest quartile (16.01-17.00%). The overall distribution of RDW-CV quartiles differed significantly between males and females (Pearson's χ² = 12.31, p = 0.006). Compared with the reference quartile (13.61-16.00%), males in the lowest RDW-CV quartile had higher odds (OR: 4.89; 95% CI: 1.30-18.42), whereas no statistically significant difference was observed for the second quartile (Table 4). Among patients with metabolic syndrome, the distribution of RDW-CV quartiles differed significantly between males and females, with females being more frequently represented in the higher RDW-CV quartiles.

**Table 5:** Distribution of RDW-CV quartiles according to gender among patients with metabolic syndrome (N = 50). *Statistically significant. RDW-CV: red cell distribution width-coefficient of variation.

RDW-CV	Males (n = 24)	Females (n = 26)	OR (95% CI)	Chi square	Degree of freedom	p-value
0-12.70	16	6	4.89 (1.30-18.42)	12.31	3	0.006*
12.71-13.60	3	4	1.42 (0.29-7.05)			
13.61-16.00	5	10	Reference			
16.00-17.00	0	6	-			

## Discussion

An elevated risk of ischemic stroke and cardiovascular disease (CVD) is linked to metabolic syndrome (MetS). Anemia has historically been assessed using RDW-CV, a frequently reported total blood count metric. Recent data, however, indicate that RDW-CV may potentially be a predictor of unfavorable cardiovascular outcomes and represents oxidative stress and chronic inflammation. Chronic obstructive lung disease, postoperative atrial fibrillation after heart surgery, albuminuria in familial Mediterranean fever, and other inflammatory conditions have all been linked to elevated RDW-CV [7-13]. The degree of variation in erythrocyte size (anisocytosis) is represented by RDW-CV, which is computed as the standard deviation of red blood cell volume divided by the mean corpuscular volume (MCV), multiplied by 100 [12,13].

The relationship between RDW-CV and metabolic syndrome has been investigated by a number of researchers; however, the results have been mixed. According to Sanchez-Chaparro et al., chronic inflammation hinders erythrocyte maturation, which leads to increased anisocytosis. They also showed that enhanced RDW-CV was independently linked to metabolic syndrome [6]. However, the majority of the research participants were made up of young, healthy, working people, which would restrict how broadly the results can be applied.

Similarly, although subgroup analysis was constrained by the comparatively small sample size, Farah and Khamisy-Farah observed that RDW-CV increased with the severity of metabolic syndrome [14]. Vayá et al. found that the only aspect of metabolic syndrome that was substantially linked to increased RDW was abdominal obesity [15]. Fujita et al., on the other hand, failed to show a substantial correlation between RDW and obesity in overweight teenagers [16]. According to Laufer Perl et al., an RDW-CV value of ≥14% was linked to a greater long-term mortality rate and a higher prevalence of metabolic syndrome [17]. This result was different from what Vayá et al. reported [15].

RDW values were higher in normotensive subjects than in hypertensive subjects, according to Emamian et al. [18]. These differences between populations may be partially explained by changes in genetic polymorphisms related to blood pressure regulation [19-23]. While Vayá et al. found no significant correlation between RDW-CV and serum triglyceride levels in a healthy population, other researchers have shown that metabolic abnormalities such as insulin resistance, hyperglycemia, and hypertriglyceridemia negatively impact erythrocyte deformability [24]. Reduced erythrocyte deformability was also linked to metabolic disorders; however, the correlation with RDW-CV was not as strong, according to Vayá et al. [25]. Therefore, increased anisocytosis seen in patients with metabolic syndrome may be related to morphological changes in erythrocytes brought on by oxidative stress.

The link between RDW-CV and metabolic syndrome may also be influenced by hyperglycemia. One of the main characteristics of metabolic syndrome is insulin resistance, which leads to poor glucose metabolism and elevated oxidative stress. A link between RDW-CV and the development of diabetes mellitus in the future was shown by Engström et al. [26]. Prolonged hyperglycemia shortens erythrocyte lifespan, decreases deformability, and modifies erythrocyte membrane characteristics, all of which increase red blood cell size variability [27,28]. The observed correlation between RDW-CV and metabolic problems may be explained by these factors.

The diverse correlations between RDW-CV and metabolic syndrome observed in various groups may possibly be attributed to genetic factors affecting susceptibility to metabolic syndrome [29].

Fifty metabolic syndrome patients, ages 18 to 60, were assessed in this study. Compared to healthy controls, patients with metabolic syndrome had considerably greater RDW-CV. While HDL cholesterol exhibited a slight negative link with RDW-CV, correlation analysis revealed significant positive relationships between RDW-CV and both belly circumference and serum triglyceride levels. However, these correlations were no longer statistically significant when participants were classified based on RDW-CV quartiles. This discrepancy is probably due to a decrease in statistical power once continuous variables were divided into quartiles.

The distribution of RDW-CV quartiles was shown to differ significantly by gender. Male participants predominated in the lower RDW-CV quartiles, while female patients were more commonly represented in the higher quartiles. However, these results should be interpreted cautiously and need confirmation in larger prospective studies due to the relatively small sample size. Yan et al. have found similar findings about gender-specific variations in the association between RDW-CV and metabolic syndrome [30].

Fasting blood glucose levels were higher in participants with metabolic syndrome, albeit this difference was not statistically significant. The fact that fasting glucose is only one component of glucose metabolism and may stay within the normal range in the early stages of insulin resistance due to compensatory hyperinsulinemia may help to explain this observation. Additionally, the inclusion of individuals undergoing antidiabetic treatment or changing their lifestyles may have lessened intergroup disparities. The relatively small sample size may have limited the statistical power of the study to detect significant changes.

Participants in the current study who had metabolic syndrome had significantly higher serum SGOT (AST) and SGPT (ALT) levels than those who did not. Given that metabolic syndrome is closely linked to non-alcoholic fatty liver disease (NAFLD), which is thought to be the hepatic manifestation of metabolic syndrome, this conclusion is medically tenable. Increased lipolysis brought on by insulin resistance causes hepatic triglyceride buildup, oxidative stress, hepatocellular damage, and excessive free fatty acid input into the liver, all of which raise liver transaminases. While moderate elevations in SGOT and SGPT often indicate underlying hepatic steatosis in those with metabolic syndrome, liver enzymes are not sensitive enough to identify NAFLD.

Limitations

There are various limitations to the current investigation. First, the statistical power may have been constrained by the small sample size, especially for subgroup analyses based on RDW-CV quartiles. Second, a statistically significant age difference between cases and controls was found, which may have affected the results even though controls were chosen to create a comparable age distribution. Third, no multivariate analysis was done to account for any confounding factors. Furthermore, the iron profile, hemoglobin A1c, inflammatory markers, and other variables that could affect RDW-CV were not evaluated. Because this was a single-center study and blood parameters were only assessed once, the results may not be as broadly applicable as they could be.

Another limitation of the present study is the use of RDW-CV as a single hematological marker of metabolic syndrome. RDW-CV was significantly increased in patients with metabolic syndrome, but RDW-CV is a global measure of erythrocyte size variability and does not adequately reflect the incremental metabolic and inflammatory burden contributed by individual components of the syndrome. The extent of insulin resistance, central obesity, hypertension, hyperglycemia, and dyslipidemia may differently influence oxidative stress, chronic inflammation, and erythropoiesis. Thus, patients with the same diagnosis of metabolic syndrome may have different degrees of systemic inflammation despite similar values of RDW-CV. In addition, RDW-CV is influenced by mean corpuscular volume and may be affected by subclinical nutritional deficiencies or other unmeasured factors. Thus, RDW-CV should be interpreted as a supportive biomarker rather than an independent marker of metabolic syndrome severity. Further studies including inflammatory markers such as high-sensitivity C-reactive protein, interleukin-6, and tumor necrosis factor-α, as well as analyses of the number and severity of metabolic syndrome components, may provide a more complete assessment of the association between anisocytosis and metabolic dysfunction. Larger multicenter prospective investigations are therefore necessary to confirm the current findings.

## Conclusions

Compared to healthy controls, patients with metabolic syndrome had noticeably higher RDW values. RDW showed a little negative link with HDL cholesterol but strong positive relationships with serum triglyceride levels and belly circumference. These results imply that RDW could be a cheap and easily accessible hematological marker that represents the metabolic and inflammatory changes linked to metabolic syndrome. 
